# Unraveling genetic etiologies in complex pediatric neurological diseases: A genetic investigation using whole exome sequencing

**DOI:** 10.1371/journal.pone.0324177

**Published:** 2025-05-19

**Authors:** Zainab Gaouzi, Aziza Belkhayat, Zahra Chebihi Takki, Hind Lachraf, Idrissa Diawara, Yamna Kriouile

**Affiliations:** 1 Mohammed VI University of Sciences and Health, Mohammed VI Higher Institute of Biosciences and Biotechnologies (UM6SS), Casablanca, Morocco; 2 Mohammed VI Center for Research and Innovation (CM6RI), Rabat Morocco; 3 BIOLAB Laboratory, Rabat, Morocco; 4 Laoratory of Microbiology and Molecular Biology/ Mohammed V University of Rabat, Rabat, Morocco; 5 Unit of Neuropediatric and Neurometabolic Diseases, Pediatrics 2, Children’s Hospital of Rabat Morocco, Faculty of Medicine and Pharmacy Rabat, University of Mohammed V Rabat, Rabat, Morocco; Shaheed Rajaei Cardiovascular Medical and Research Center: Rajaie Cardiovascular Medical and Research Center, IRAN, ISLAMIC REPUBLIC OF IRAN

## Abstract

Pediatric neurological disorders are a diverse group of conditions affecting the nervous system in children, often challenging to diagnose due to their nonspecific and overlapping clinical features. Advances in molecular diagnostics, particularly whole exome sequencing (WES), have significantly improved the identification of genetic causes, enabling precise diagnoses and personalized treatments. This study explores the application of WES in diagnosing pediatric neurological disorders within Moroccan childrens with undiagnosed or challenging pediatric neurological conditions to uncover genetic causes of complex pediatric neurological conditions unresolvable by traditional diagnostic methods. The study included 188 pediatric patients with complex neurological conditions from the Children’s Hospital of Rabat who underwent exome sequencing to investigate suspected genetic causes. WES revealed a diagnostic yield of 45%, identifying conditions such as intellectual disabilities, hereditary metabolic disorders and epilepsies. It also uncovered neurodevelopmental and neurodegenerative disorders, neuromuscular diseases, and genetic syndromes. A total of 157 variants were detected: 34% were classified as pathogenic, 28.5% as likely pathogenic, and 37.5% as variants of uncertain significance (VUS). These findings underscore the utility of WES as a robust diagnostic tool, providing insights into genetic causes and enabling tailored treatment strategies. They also highlight the importance of expanding genetic research to improve diagnostic accuracy and clinical management of pediatric neurological disorders.

## Introduction

Pediatric Neurological Disorders encompass a spectrum of conditions affecting the nervous system in children, characterized by diverse symptoms and complex presentations [1]. These disorders pose significant challenges in diagnosis and treatment due to their varied manifestations and the limited understanding of their underlying genetic causes [2].

Diagnosing Pediatric Neurological Disorders is often fraught with difficulties. Symptoms can be nonspecific or overlap with other conditions, leading to misdiagnosis or delayed diagnosis [3]. Moreover, many of these rare disorders further complicate the diagnostic process. Traditional diagnostic approaches often struggle to provide precise molecular diagnoses, hindering prognostic accuracy and targeted therapeutic interventions. However, advancements in molecular diagnostic technologies, such as next-generation sequencing (NGS), offer promising avenues for elucidating the genetic basis of these conditions [4].

Whole exome sequencing (WES), a specializes subset of NGS, has emerged as a valuable tool in identifying genetic causes of Pediatric Neurological Disorders by efficiently sequencing the protein-coding regions of the genome where a majority of disease-causing mutations reside [5]. Its application has greatly facilitated the rapid identification of genetic etiologies, allowing for the development of tailored therapeutic strategies and enhancing prognostic capabilities [6].

For this study, we enrolled cases where traditional diagnostic approaches fell short, either due to elusive symptoms or resistance to treatment. In response to these diagnostic impasses, we turned to WES as a promising avenue for dissecting the genetic underpinnings of these complex conditions. By integrating detailed clinical histories with advanced sequencing techniques, we aimed to identify pathogenic mutations underlying the observed phenotypes.

## Materials and methods

### Study participant population

The study included 188 patients treated at the neuropediatric services of the Children’s Hospital of Rabat of the University Hospital Center, presenting complex neurological diseases suspected to have a genetic etiology, or faced diagnostic challenges including resistance to conventional treatments from 12-05-2017 to 15-11-2023. Exome sequencing was conducted on all patients as probands, after obtaining informed consent from their guardians. In cases where the proband’s exome sequencing results were positive, and parents were willing to investigate the genetic disorder’s inheritance, further examination proceeded with Sanger sequencing of the parents. Ethical approval for this study was obtained from the Ethics Committee for Biomedical Research of the Faculty of Medicine and Pharmacy of Rabat n°35/17.

### Study design

**Fig 1:**
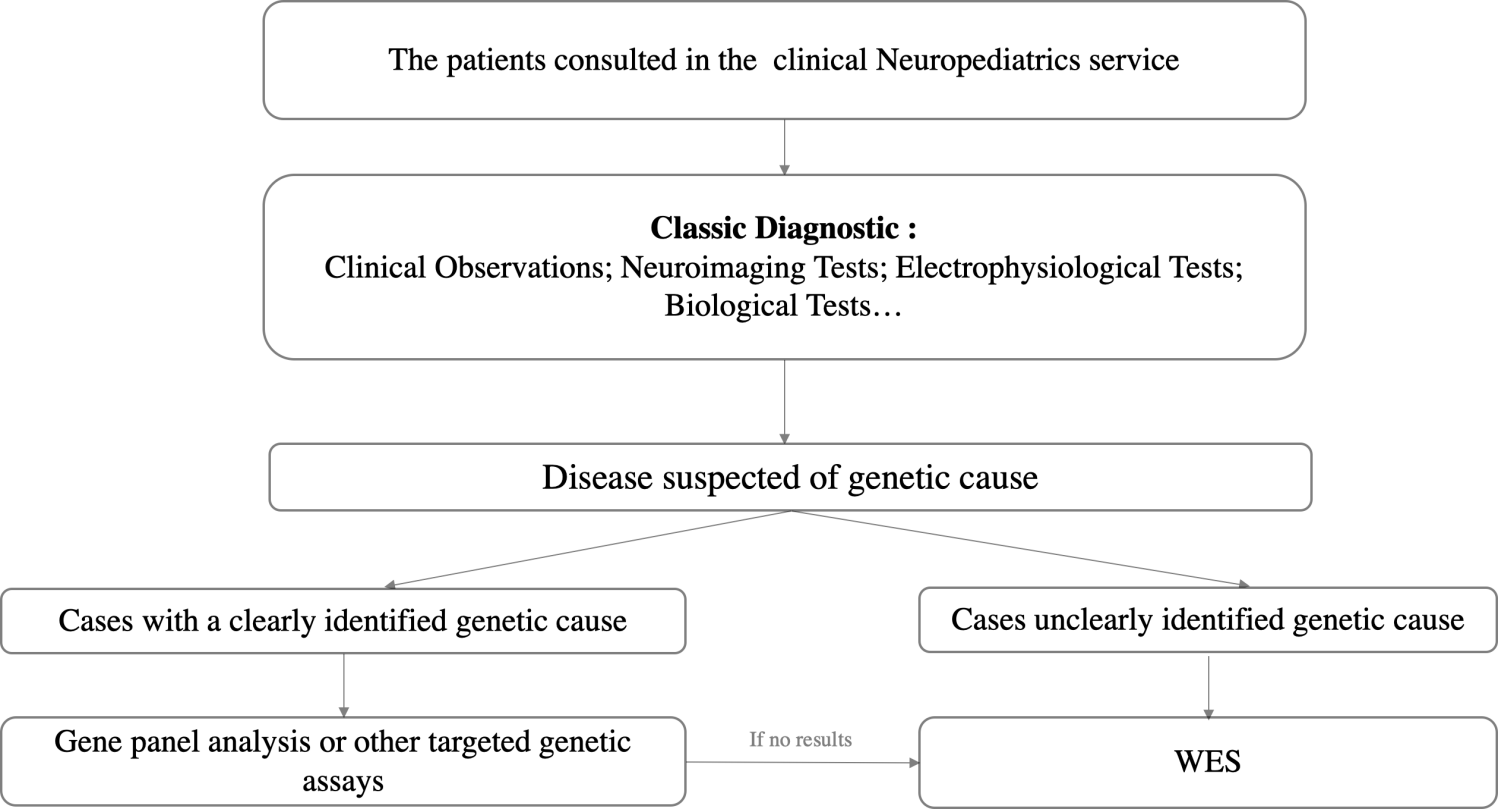
Workflow for genetic investigation in pediatric neurological disorders.

At the neuropediatrics service of the Children’s Hospital of rabat, experienced neuropediatricians diagnose and manage a wide range of pediatric neurological disorders annually. The aim is to deliver the optimal diagnosis and treatment for each patient, tailored to their circumstances. This process often entails thorough diagnostic assessments, including magnetic resonance imaging (MRI) scans, comprehensive laboratory evaluations, and additional auxiliary investigations. However, certain patients encounter diagnostic challenges or demonstrate resistance to conventional treatments, resulting in diagnostic and therapeutic dilemmas. Among these cases, where the healthcare professionals (neuropediatrician and geneticist) suspect a potential genetic etiology for the patient’s conditions, WES is employed to explore the underlying genetic basis. It is worth noting that cases with an identified genetic cause undergo exclusion from exome sequencing, as they typically undergo basic genetic tests such as gene panel analysis or other targeted genetic assays. When these tests yield negative results, WES is then performed (Fig 1). In this study, we focus solely on the results obtained through WES.

### Whole exome sequencing

WES was conducted in partnership with the laboratories Cerba (Frepillon, France) and Centogene (Rostock, Germany), ensuring a high-quality and collaborative approach throughout the process. DNA extraction was performed using the QIAamp DNA Blood Mini Kit (Qiagen, Valencia, CA, USA) following the manufacturer’s protocol. Extracted DNA samples were then stored at -20°C until sequencing.

Exome sequencing was performed on patient samples using a comprehensive method involving the enrichment of exomic regions of the human genome. Followed by paired-end sequencing reactions. This process utilized capture baits targeting approximately 36.5 Mb of the human coding exome using Illumina’s Nextera Rapid Capture Exome Kit, SureSelect Human All Exon V5 kit, or Twist Human Core Exome, covering over 98% of coding RefSeq from the human genome build GRCh37/hg19. The enriched target regions from fragmented genomic DNA were sequenced using NovaSeq 6000, NextSeq500 sequencers, or HiSeq 2000 (Illumina), ensuring at least 20x coverage depth for more than 98% of targeted bases. The sequencing data underwent analysis through a computational pipeline that included read alignment to the GRCh37/hg19 of the human reference genome using the Burrows-Wheeler Aligner (BWA) [7]. The resulting alignments were converted into binary BAM file format, sorted on the fly, and de-duplicated to remove PCR duplicates. The Genome Analysis Toolkit (GATK) pipeline was used to refine alignments, including recalibrating base quality scores (BQSR) and marking duplicates. Subsequently, variant calling was performed on the secondary alignment files using GATK HaplotypeCaller, which was used to identify single nucleotide variants (SNVs), and Manta [8] was used for detecting copy number variants (CNVs). The identified variants were then annotated using Annovar [9]. Confirmation of parental variants was conducted using Sanger sequencing when parental DNA was available. Low-quality variants were validated through Sanger sequencing, quantitative polymerase chain reaction (qPCR), or microarray technology (CytoScan® 750K, Array Array-CGH).

### Variant interpretation

Interpretation of genetic variants involved establishing an analytical framework guided by specific criteria. This included identifying variations impacting protein sequences or canonical splice sites with frequencies lower than 1%, as determined by data from the dbSNP [10] and gnomAD databases, as well as variants reported as disease-causing in databases such as HGMD, ClinVar [11], or CentoMD. The primary goal of genetic variation interpretation was to identify clinically relevant variants validated through independent methodologies, focusing on genes relevant to the patient’s condition as determined by the attending clinician. The investigation prioritized variants within coding exons and adjacent intronic regions of genes with well-established gene-phenotype correlations sourced from OMIM information. Various inheritance patterns were evaluated, incorporating familial history and clinical data to ascertain the pathogenicity and causality of identified variants. Classification of variants followed the ACMG guidelines [12], categorizing them as pathogenic, likely pathogenic, variants of uncertain significance (VUS), likely benign, or benign. All relevant variants were thoroughly documented in relation to the patient’s phenotype.

### Statistical analysis

Descriptive statistics summarized demographic and clinical characteristics of the patients. Associations between clinical features and the individuals with positive WES results were examined using linear and logistic regression models, depending on the nature of the outcome variable. The RStudio software was employed for all statistical analyses, setting significance at a p-value below 0.05. Results, including odds ratios and confidence intervals, were visualized using the ggplot2 package.

### Pathway analysis

We used the STRING database [13]. The list of genes identified in the study was uploaded to STRING for analysis. We set the interaction confidence threshold to high (≥0.7) to ensure robust results and included both direct and indirect interactions. Functional enrichment analysis was conducted, focusing on KEGG pathways [14], Reactome pathways [15], and Gene Ontology (GO) terms [16]. This approach aimed to uncover key molecular and cellular processes associated with the genetic variants observed in the study.

### Circular plot construction

We used the R programming language with the “circlize” and “RColorBrewer” packages. Data on chromosome sizes were downloaded from the human reference genome GRCh37/hg19, and gene positions were obtained from the same reference genome using the Ensembl BioMart database. A set of genes of interest was queried, and their chromosome locations were retrieved, filtered to retain only standard chromosomes (1–22, X, and Y), and formatted for further analysis. Gene sizes were calculated from genomic coordinates and normalized to adjust line thickness. Each gene was assigned a distinct color for clear visualization. Mutation data were integrated, with specific colors representing different mutation types. The final visualization was created using “circlize”.

## Results

### Characteristics of the patients

The study characterization includes a diverse sample of 188 individuals, with an average age at genetic testing of approximately 5.80 years (SD ± 4.08), ranging from 4 month to 18 years. The study population exhibits a slight male predominance, with 101 males (53.7%) compared to 87 females (46.3%). 36.2% of the participants come from consanguineous unions, indicating a strong potential for familial genetic linkage, and 28.7% have a family history of the same condition. In terms of clinical features, 71.3% of individuals exhibited motor delays, 63.3% have intellectual disabilities, 58% have language disorders, 43.1% experience epileptic seizures, and 32.4% present hypotonia. Other notable features included Attention-Deficit/Hyperactivity Disorder (ADHD) is present in 26.1% of the subjects, while Facial dysmorphia is observed in 17.6% of the studied group, and Autism Spectrum Disorder (ASD) in 16% (Table 1).

**Table 1:** Demographic and Clinical Characteristics of patients with or without a genetic variant.

	Group Without Genetic variants (N = 60)	Group With Genetic variants (N = 128)	Overall (N = 188)
**Age at testing (years)**			
Mean (SD)	6.08 (3.93)	5.67 (4.16)	5.80 (4.08)
Median [Min, Max]	6.00 [0.500, 16.0]	5.00 [0.330, 18.0]	5.00 [0.330, 18.0]
**Gender**			
F	25 (41.7%)	62 (48.4%)	87 (46.3%)
M	35 (58.3%)	66 (51.6%)	101 (53.7%)
**Consanguineous**			
Consanguineous	38 (63.3%)	76 (59.4%)	114 (60.6%)
Non consanguineous	18 (30.0%)	50 (39.1%)	68 (36.2%)
Missing	4 (6.7%)	2 (1.6%)	6 (3.2%)
**Family history**			
No	45 (75.0%)	88 (68.8%)	133 (70.7%)
Yes	14 (23.3%)	40 (31.3%)	54 (28.7%)
Missing	1 (1.7%)	0 (0%)	1 (0.5%)
**Motor delay**			
No	18 (30.0%)	36 (28.1%)	54 (28.7%)
Yes	42 (70.0%)	92 (71.9%)	134 (71.3%)
**Intellectual disability**			
No	24 (40.0%)	45 (35.2%)	69 (36.7%)
Yes	36 (60.0%)	83 (64.8%)	119 (63.3%)
**Language disorder**			
No	20 (33.3%)	59 (46.1%)	79 (42.0%)
Yes	40 (66.7%)	69 (53.9%)	109 (58.0%)
**Epileptic seizures**			
No	35 (58.3%)	72 (56.3%)	107 (56.9%)
Yes	25 (41.7%)	56 (43.8%)	81 (43.1%)
**Hypotonia**			
No	38 (63.3%)	89 (69.5%)	127 (67.6%)
Yes	22 (36.7%)	39 (30.5%)	61 (32.4%)
**ADHD**			
No	43 (71.7%)	96 (75.0%)	139 (73.9%)
Yes	17 (28.3%)	32 (25.0%)	49 (26.1%)
**Facial dysmorphia**			
No	48 (80.0%)	107 (83.6%)	155 (82.4%)
Yes	12 (20.0%)	21 (16.4%)	33 (17.6%)
**ASD**			
No	48 (80.0%)	110 (85.9%)	158 (84.0%)
Yes	12 (20.0%)	18 (14.1%)	30 (16.0%)

*ADHD: Attention-Deficit/Hyperactivity Disorder; ASD: Autism Spectrum Disorder.

S1 Table summarizes the patient’s epidemiologic, clinical, and Paraclinical data. All patients exhibited various neurological manifestations such as seizures, developmental delay, intellectual disability, facial dysmorphism, visual impairment, hearing abnormalities, and abnormal movements. Diagnostic investigations, such as brain MRI, computerized tomography (CT) scans, electroencephalogram (EEG), electroneuromyography (ENMG), and electromyography (EMG) were performed based on clinical indications. Neuroimaging and neurophysiological findings varied widely among patients. MRI results ranged from normal to showing specific abnormalities, such as leukodystrophy, cortical atrophy, and ventricular ectasia. Additional findings included both cortical and subcortical atrophy, as well as signs suggestive of mitochondrial diseases and metabolic disorders. EEG findings showed a wide range of outcomes, including normal readings, benign partial epilepsy in infancy, and the absence of epileptic anomalies. Some cases revealed diffuse cerebral suffering and EEG abnormalities, such as interictal epileptic anomalies and rare right frontotemporal spike discharges. (S1 Table) also includes the initial clinical suspicions that were reached based on comprehensive clinical and paraclinical evaluations. These preliminary diagnoses, formulated by pediatric neurologists, integrated presenting symptoms, physical examination findings, and supporting investigations.

### Exome sequencing results

#### Overview of genetic diagnoses.

Out of the 188 individuals, 60 (32%) had negative results, with no pathogenic variants identified. Among these negative cases, incidental findings were reported in two individuals, consisting of two SNVs. These variants included a KCNQ1 variant (c.914G > T, p.Trp305Leu) associated with Long QT syndrome 1 (proband 87), and an MEFV variant (c.2082G > A, p.Met694Ile) associated with Familial Mediterranean fever, autosomal dominant (proband 113).

In contrast, genetic variants were identified in 128 individuals, and classified the WES results as either pathogenic, likely pathogenic or VUS. Among these 128 individuals, 85 patients (45%) had a conclusive positive diagnostic result. Notably, this group included 11 patients who carried two different pathogenic or likely pathogenic variants, seven had both a pathogenic or likely pathogenic variant combined with a VUS, and 1 patient carried two pathogenic variants in addition to a VUS. Additionally two patients had CNVs, who also received conclusive diagnoses. One patient harbored two different CNVs, while another carried a CNV along with a VUS. These CNVs included two chromosomal microdeletions and one microduplication on chromosomes 6, 9 and 16. The size of the involved regions ranged from 1.3 to 17.23 Mb (Table 2).

**Table 2:** Summary of CNVs identified in neuropediatric patients.

CNV					
Patient ID	Deletion/ Duplication	Location	Classification	Estimated Size (Mb)	Name of disease
84	Deletion	6q27	P	3.73	Brain malformations.
	Duplication	16p13.3p12.3	P	17.23	Intellectual disability and congenital anomalies.
118	Deletion	9q33.3	P	1.3	Autosomal dominant early infantile epileptic encephalopathy type 4.

The remaining 43 patients (23%) carried only VUS, including seven patients harboring two distinct VUS. The phenotype-genotype correlation revealed that 30 out of these 43 patients had phenotypes consistent with the identified genetic VUS variants. However, the remaining cases showed discrepancies, including three patients where the phenotype could not be conclusively explained due to conflicting traits that both supported and contradicted the effects of the genetic mutation.

#### Association of clinical factors with the presence of genetic variant.

**Fig 2:**
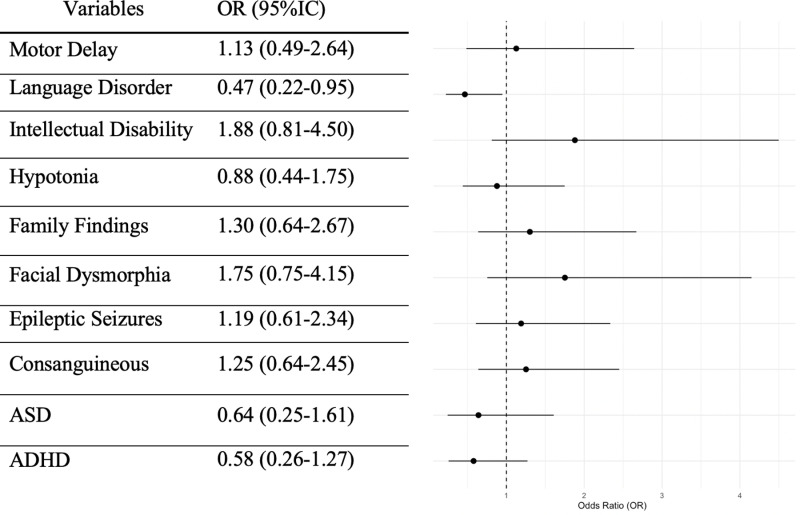
Logistic regression analysis of whole exome sequencing data.

Ten phenotypic variables were evaluated for their association with individuals that had a conclusive positive diagnostic result using logistic regression analysis. The model revealed that language disorder has a statistically significant association with a reduced probability of detecting a genetic variant, with an odds ratio (OR) of 0.48 (95% CI: 0.23-0.97). Other factors, such as ASD and ADHD, showed a less reduced probability of detecting a genetic variant, with ORs close to 1 and wider confidence intervals. Intellectual disability was associated with a higher likelihood of the outcome, with an OR of 1.86, although the wide confidence interval suggested uncertainty in the estimate. Facial dysmorphia, family history and consanguinity also showed increased odds with ORs of approximately 1.70, 1.26 and 1.28, respectively, though their CIs overlapped with 1, indicating these associations are not statistically significant. Motor delay, hypotonia and epileptic seizures exhibited ORs close to 1, reflecting weaker or no significant associations with the outcome (Fig 2).

#### Variants finding analysis.

Overall, the analysis identified a total of 157 variants, including 3 CNVs and 154 SNVs. However, among the SNVs, three variants were recurrent: one appeared three times, while two others were found twice (Table 3). According to the ACMG classification system, 50 variants (32%) were classified as pathogenic, 45 (29.5%) as likely pathogenic, and 59 (38.5%) as VUS.

**Table 3:** Summary of SNV identified in neuropediatric patients by WES.

SNV									
Patient ID	Gene	cDNA changes	Variants	Classification	Zygosity	Inheritance	Name of disease/OMIM discription	Literature Reported Variants	ClinVar Reported Variants
1	LAMA2	NM_000426.4	c.8244 + 1G > A	P	Het		LAMA2-related muscular dystrophy	Described	Described
			c.5290dup (p.Glu1764GlyfsTer3)	P	Het		LAMA2-related muscular dystrophy	Described	Described
3	QDPR	NM_000320.3	c.323G > A (p.Trp108Ter)	P	Hom		Hyperphenylalaninemia, Bh4-Deficient	Not Described	Not Described
	CFTR	NM_000492.4	c.3484C > T (p.Arg1162Ter)	P	Het		Cystic Fibrosis	Described	Described
5	DONSON	NM_017613.4	c.82A > C (p.Ser28Arg)	LP	Het		Microcephaly-micromelia syndrome	Described	Described
9	TUBB4A	NM_006087.4	c.535G > T (p.Val179Leu)	LP	Het		Leukodystrophy, Hypomyelinating	Not Described	Not Described
13	SYNGAP1	NM_006772.3	c.1685C > T (p.Pro562Leu)	LP	Het		SYNGAP1-related developmental and epileptic encephalopathy	Described	Described
16	SLC16A2	NM_006517.5	c.1343_1344dup (p.Gly449LeufsTer7)	LP	Hem		Allan-Herndon-Dudley Syndrome	Not Described	Not Described
17	KCNMA1	NM_001161352.2	c.2267-2A > T	LP	Het		Epilepsy, idiopathic generalized, susceptibility to paroxysmal nonkinesigenic dyskinesia, with or without generalized epilepsy	Not Described	Not Described
18	CTNNB1	NM_001904.4	c.998dup (p.Tyr333Ter)	LP	Het		Neurodevelopmental disorder with spastic diplegia and visual	Described	Described
19	SH3TC2	NM_024577.4	c.3325C > T (p.Arg1109Ter)	P	Het		Charcot-Marie-Tooth disease type 4C	Described	Described
			c.2595_2596dup (p.Ala866GlufsTer21)	LP	Het		Charcot-Marie-Tooth disease type 4C	Not Described	Not Described
20	NKX2–1	NM_001079668.3	c.586del (p.Val 196 CysfsTer32)	LP	Het	AD	Choreoathesis, hypothyrodism and neonatal respiratorydis	Not Described	Not Described
26	MEF2C	NM_002397.5	c.402 + 1G > A	LP	Het	AD	Intellectual disability, autosomal dominant 20	Not Described	Described
28	GCDH	NM_000159.4	c.1213A > G (p.Met405Val)	P	Hom	AR	Déficit en glutaryl-CoA déshydrogénase	Described	Described
29	ABHD16A	NM_021160.2	c.1267_1268delinsG (p.Leu423Glyfs*2)	VUS	Hom		Complex Hereditary Spastic Paraplegia	Not Described	Not Described
30	KMT2B	NM_014727.3	c.4203dup (p.Arg 1402AlafsTer276)	P	Het	DE NOVO	Dystonia 28, Chilhood-onset	Not Described	Not Described
31	WWOX	NM_016373.4	c.403G > C (p.Gly135Arg)	LP	Hom	AR	Nonspecific early-onset epileptic encephalopathy	Not Described	Not Described
	TOR1A	NM_000113.3	c.907_909del (p.Glu303del)	P	Het	DE NOVO	Early-onset generalized limb-onset dystonia	Described	Described
34	SLC2A1	NM_006516.4	c.101A > G (p.Asn34Ser)	P	Het	AD	Paroxysmal dystonic choreoathetosis with epileptic ataxia Autosomal dominant	Described	Described
35	RNASEH2C	NM_032193.4	c.451C > T (p.Pro151Ser)	LP	Hom	AR	Aicardi-Goutieres syndrome 3	Described	Described
37	HEXA	NM_000520.6	c.62del (p.Thr21ArgfsTer79)	LP	Hom	AR	Tay-Sachs disease	Not Described	Not Described
38	ATP1A3	NM_152296.5	c.2452G > A (p.Glu818Lys)	LP	Het	AR	Alternating hemiplegia of childhood	Described	Described
39	DHCR7	NM_001360.3	c.964-1G > C	P	Het	AR	Smith-Lemli-Opitz syndrome	Described	Described
			c.506C > T (p.Ser169Leu)	LP	Het	AR	Smith-Lemli-Opitz syndrome	Described	Described
40	RNASEH2B	NM_024570.4	c.529G > A (p.Ala177Thr)	P	Hom	AR	Aicardi-Goutieres syndrome 2	Described	Described
41	CTSA	NM_000308.4	c.743T > G (p.Phe24 8Cys)	LP	Hom	AR	Galactosialidosis	Not Described	Not Described
44	COLQ	NM_005677.4	c.1193T > A (p.Ile39 8Asn)	VUS	Hom	AR	Congenital myasthenic syndrome	Not Described	Not Described
45	ASPM	NM_018136.5	c.4195dup (p.Thr1399AsnfsTer20)	P	Hom		Primary autosomal recessive microcephaly	Described	Described
46	DENND5A	NM_015213.4	c.127C > T (p.Gln43Ter)	P	Hom	AR	Developmental and epileptic encephalopathy 49	Described	Described
47	ERCC2	NM_000400.3	c.1838A > G (p.His613Arg)	LP	Hom		Spectrum of anomalies associated with the ERCC2 gene	Not Described	Described
48	TRAPPC9	NM_001374682.1	c.1099C > T (p.Arg367Trp)	VUS	Hom	AR	Intellectual Developmental Disorder	Not Described	Described
	PIGO	NM_032634.4	c.1132C > T (p.Leu378Phe)	VUS	Hom	AR	Hyperphosphatasia-Intellectual Disability Syndrome	Described	Described
49	COL13A1	NM_001368882.1	c.118C > T (p.Pro40Ser)	VUS	Hom		Synaptic congenital myasthenic syndromes	Not Described	Described
50	AFG3L2	NM_006796.3	c.916A > G (p.Lys306Glu)	LP	Het	AR	Spastic ataxia-myoclonic epilepsy-neuropathy syndrome	Not Described	Described
			c.1885_1886del (p.Leu629ArgfsTer6)	LP	Het	AR	Spastic ataxia-myoclonic epilepsy-neuropathy syndrome	Not Described	Not Described
52	PRMT7	NM_019023.4	c.1544C > A (p.Ala515Asp)	LP	Hom	AR	Short stature-brachydactyly-obesity-retardation syndrome	Not Described	Not Described
54	LAMA2	NM_000426.3	c.9106del (p.Arg3036AlafsTer43)	LP	Hom	AR	Alpha subunit-related congenital muscular dystrophy	Not Described	Not Described
55	NPC1	NM_000271.5	c.3120_3122delinsTT (p.Tyr1041SerfsTer15)	LP	Het	AR	Niemann-Pick disease	Not Described	Not Described
			c.2830G > A (p.Asp944Asn)	P	Het	AR	Niemann-Pick disease	Not Described	Not Described
56	TUBB4A	NM_006087.4	c.50G > A (p.Gly17Glu)	P	Het	DE NOVO	Hypomyelination with atrophy of the central gray nuclei	Not Described	Not Described
57	FGF14	NM_004115.4	c.409-2A > G	P	Het	AD	Spinocerebellar ataxia type 27 Autosomal	Not Described	Not Described
59	MFSD8	NM_001371595.1	c.831del (p.Phe277LeufsTer2)	LP	Het	AR	Neuronal Ceroid-lipofuscinosis late childhood	Described	Described
			c.530C > T (p.Ala177Val)	VUS	Het	AR	Neuronal Ceroid-lipofuscinosis late childhood	Not Described	Not Described
60	TH	NM_000360.3	c.1229G > C (p.Arg410Pro)	LP	Het	AR	Autosomal recessive dopa-responsive dystonia	Described	Described
			c.883T > C (p.Ser295Pro)	LP	Het	AR	Autosomal recessive dopa-responsive dystonia	Not Described	Not Described
62	CNGA3	NM_001298.3	c.1114C > T (p.Pro372Ser)	P	Hom	AR	Achromatopsia 2	Described	Described
63	RNASEH2B	NM_024570.4	c.529G > A (p.Ala177Thr)	P	Hom	AR	Aicardi-Goutieres syndrome 2	Described	Described
64	COL1A1	NM_000088.4	c.3226G > A (p.G1076S)	P	Het	AR	Osteogenesis imperfecta	Described	Described
66	EIF2B3	NM_020365.5	c.32G > T (p.Gly11Val)	LP	Hom	AR	Leukoencephalopathy with vanishing white matter	Described	Described
69	PIGN	NM_012327.5	c.2791_2794del (p.M931EfsTer18)	VUS	Hom	AR	Multiple congenital anomalies-hypotonia-epileptic syndrome	Not Described	Not Described
70	TPP1	NM_000391.4	c.617G > A (p.Arg206His)	P	Hom	AR	congenital multiple-hypotonia-epileptic	Described	Described
73	IRF2BPL	NM_024496.4	c.514G > T (p.E172Ter)	LP	Het	DE NOVO	Neurodevelopmental disorder with regression, abnormalmovement	Described	Described
74	CSNK2A1	NM_001895.3	c.209T > A (p.L70H)	LP	Het	DE NOVO	Okur-Chung neurodevelopmental Syndrome	Not Described	Not Described
75	KRAS	NM_004985.5	c.40G > A (p.Val14Ile)	P	Het		Noonan syndrome 3	Described	Described
76	ITGA7	NM_001144996.2	c.1100dup (p.His368Serfs*15)	P	Hom		Congenital muscular dystrophy due to integrin alpha-7 deficiency	Described	Described
78	PCCA	NM_000282.4	c.317C > A (p.Ala106Glu)	P	Hom		Propionic acidemia	Described	Described
79	ADD3	NM_001320591.1	c.1100G > A (p.Gly367Asp)	P	Hom		Spastic Tetraplegia Cerebral Palsy Type 3	Described	Described
80	GJC2	NM_020435.3	c.816C > A (p.Tyr272*)	LP	Hom		Pelizaeus-Merzbacher-like type 1 disease, spastic paraplegia 44, autosomal dominant lymphatic malformation	Not Described	Not Described
81	HIVEP2	NM_006734.3	c.3813_3814delinsCA (p.Ala1272Thr)	VUS	Het		Mental retardation type 43	Not Described	Not Described
82	IQSEC2	NM_001111125.2	c.2911C > T (p.Arg971*)	LP	Het		Intellectual disability, X-linked 1	Described	Described
	IRF2BPL	NM_024496.3	c.1843T > C (p.Ser615Pro)	VUS	Het		Neurodevelopmental Disorder With Regression, Abnormal Movements, Loss Of Speech, And Seizures	Not Described	Not Described
87	SCN1A	NM_001202435.2	c.5341T > A (p.Tyr1781Asn)	P	Het		SCN1A-related seizure disorders	Not Described	Not Described
88	FRRS1L	NM_014334.3	c.436dup (p.Ile146Asnfs*10)	P	Hom		Early infantile epileptic encephalopathy type 37	Described	Not Described
90	CNTNAP2	NM_014141.6	c.550 + 5G > A	VUS	Hom		CNTNAP2-Related Disorder: Pitt-Hopkins Syndrome- Cortical Dysplasia Syndrome Focal Epilepsy	Described	Described
91	PTPN11	NM_001330437.1	c.188A > G (p.Tyr63Cys)	P	Het		Noonan syndrome 1	Described	Described
92	STXBP1	NM_003165.3	c.364C > T (p.Arg122*)	P	Het		Developmental and epileptic encephalopathy, 4	Described	Described
93	PLA2G6	NM_003560.2	c.2234G > A (p.Arg745Gln)	VUS	Het		Infantile neuroaxonal dystrophy	Not Described	Described
			c.1442T > A (p.Leu481GIn)	P	Het		Infantile neuroaxonal dystrophy	Described	Described
			c.1933C > T (p.Arg645*)	P	Het		Infantile neuroaxonal dystrophy	Described	Described
94	PEX2	NM_000318.2	c.88G > T (p.Glu30*)	LP	Het		Disorder of the biogenesis of peroxisomes 5A or 5B	Not Described	Not Described
			c.750G > A (p.Trp250*)	LP	Het		Disorder of the biogenesis of peroxisomes 5A or 5B	Not Described	Not Described
97	SATB2	NM_001172509.1	c.474-2A > G	LP	Het		Chromosome 2q32-q33 deletion syndrome	Not Described	Described
99	CFTR	NM_000492.4	c.3808G > A (p.Asp1270Asn)	P	Het		CFTR-related disorder	Described	Described
			c.220C > T (p.Arg74Trp)	P	Het		CFTR-related disorder	Described	Described
100	SYNE1	NM_182961.2	c.3368C > G (p.Pro1123Arg)	VUS	Het		Emery-Dreifuss muscular dystrophy type 4	Not Described	Not Described
102	SLC6A1	NM_001348250.1	c.487_488del (p.Gln163Valfs*43)	LP	Het		Epilepsy myoclonique-atonique	Not Described	Not Described
103	LPIN1	NM_001261428.3	c.2570C > G (p.Pro857Arg)	VUS	Hom		Acute recurrent myoglobinuria	Not Described	Described
104	RAC1	NM_018890.3	c.191A > G (p.Tyr64Cys)	LP	Het		Intellectual disability, autosomal dominant 48;	Described	Described
	POGZ	NM_ 015100.3	c.941G > A (p.Ser314Asn)	VUS	Het		The genetic diagnosis of autosomal dominant White-Sutton syndrome is possible.	Not Described	Not Described
105	FRMPD4	NM_014728.3	c.159T > G (p.Asn53Lys)	VUS	Hem		X-Related Mental Retardation Type 104	Not Described	Not Described
107	PLA2G6	NM_003560.4	c.1442T > A (p.Leu481Gln)	P	Het		Infantile neuroaxonal dystrophy	Described	Described
108	PPT1	NM_000310.3	c.490C > T (p.Arg164*)	P	Hom	AR	Neuronal ceroid lipofuscinosis 1	Described	Described
109	IQSEC2	NM_001111125.3	c.3279G > A (p.Ser1093Ser)	VUS	Hem		Intellectual disability, X-linked 1	Described	Described
110	GRM1	NM_001278064.1	c.277C > T (p.Pro93Ser)	VUS	Het		Spinocerebellar ataxia 44	Not Described	Not Described
113	AP4M1	NM_004722.4	c.1137 + 1G > T	P	Hom		Autosomal recessive spastic paraplegia type 50	Described	Described
114	SPG11	NM_025137.3	c.4365G > C (p.Trp1455Cys)	VUS	Hom		Allan-Herndon-Dudley Syndrome	Described	Described
115	ITPR1	NM_001168272.2	c.7748T > C (p.Ile2583Thr)	VUS	Het		ITPR1-associated cerebellar ataxia spectrum disorder	Described	Described
117	IFIH1	NM_ 022168.3	c.769 + 1G > T	VUS	Het		Singleton-Merten Dysplasia	Not Described	Not Described
118	CHAMP1	NM_001164144.2	c.751G > A (p.Ala251Thr)	VUS	Het		Autosomal dominant mental retardation type 40	Not Described	Not Described
119	KCNA2	NM_004974.3	c.1228G > T (p.Val410Leu)	VUS	Het	DE NOVO	Early infantile epileptic encephalopathy type 32	Not Described	Not Described
120	SIM1	NM_005068.3	c.554G > A (p.Cys185Tyr)	VUS	Het		Gene associated with severe obesity	Not Described	Not Described
122	COL6A1	NM_001848.2	c.837_854del (p.Glu279_Gly284del)	VUS	Het		Collagen Vi-Related Congenital Muscular Dystrophy	Not Described	Not Described
			c.2782C > T (p.Arg928Cys)	VUS	Het		Collagen Vi-Related Congenital Muscular Dystrophy	Not Described	Not Described
123	FOXP2	NM_148898.3	c.665C > T (p.Ala222Val)	VUS	Het		Language and speech disorders	Not Described	Not Described
125	RNASEH2B	NM_024570.3	c.529G > A (p.Ala177Thr)	P	Hom		Aicardi-Goutieres syndrome 2	Described	Described
126	SCN2A	NM_001040142.1	c.4841T > C (p.Leu1614Pro)	VUS	Het		Developmental and epileptic encephalopathy, 11	Not Described	Described
	PHIP	NM_017934.6	c.1223C > T (p.Thr408lle)	VUS	Het		Chung-Jansen Syndrome	Not Described	Not Described
127	CLN6	NM_017882.2	c.177C > G (p.Asp59Glu)	VUS	Hom	AR	Neuronal Ceroid Lipofuscinosis Type 6	Not Described	Not Described
128	TCF4	NM_001243226.3	c.1296G > A (p.Ser432Ser)	LP	Het	AR	TCF4 Related Disorder: Pitt-Hopkins Syndrome - Fuch Corneal Endothelial Dystrophy Type 3	Described	Described
129	KMT2D	NM_003482.4	c.12545G > T (p.Gly4182Val	VUS	Het		Kabuki syndrome type 1	Not Described	Described
130	OTC	NM_000531.6	c.386G > A (p.Arg129His)	P	Het		Ornithine transcarbamylase (OTC) deficiency	Described	Described
132	HEXA	NM_001318825.2	c.566G > A (p.Arg189His)	P	Hom		Tay-Sachs disease	Not Described	Not Described
134	LMNA	NM_170707.2	c.1234G > A (p.Gly412Arg)	VUS	Het		Charcot-Marie-Tooth disease type 2	Not Described	Described
135	OCRL	NM_001318784.1	c.1621C > T (p.Arg541Ter)	P	Hem		Lowe syndrome	Described	Described
	AUTS2	NM_015570.4	c.533G > A (p.Gly178Glu)	VUS	Het		Intellectual Developmental Disorder, Autosomal Dominant 26	Not Described	Not Described
136	SEPSECS	NM_016955.4	c.114 + 3A > G	VUS	Hom		Pontoneocerebellar hypoplasia	Described	Described
137	STXBP1	NM_003165.3	c.1095_1096del (p.Cys366Profs*13)	P	Het		Early Autosomal Dominant Infantile Epileptic Encephalopathy Type 4	Not Described	Described
138	GLI2	NM_005270.4	c.595G > C (p.Gly199Arg)	VUS	Het		Autosomal dominant GLI2-related disorder	Not Described	Not Described
	DNM1	NM_001288739.1	c.1076G > A (p.Gly359Glu)	LP	Het		Developmental and epileptic encephalopathy, 31	Not Described	Described
139	MAT1A	NM_000429.2	c.790C > T (p.Arg264Cys)	LP	Hom		Autosomal recessive methionine adenosyltransferase deficiency.	Described	Described
	C12orf57	NM_001301834.1	c.1A > G (p.Met1Val)	P	Hom		Temtamy syndrome	Described	Described
140	ABCD1	NM_000033.3	c.338G > T (p.Arg113Leu)	VUS	Hem		X-linked adrenoleukodystrophy	Not Described	Described
			c.1508T > C (p.Leu503Pro)	VUS	Hem		X-linked adrenoleukodystrophy	Not Described	Described
141	PACS1	NM_018026.4	c.607C > T (p.Arg203Trp)	P	Het		PACS1-related syndrome	Described	Described
142	FKRP	NM_000033.3	c.1364C > A (p.Ala455Asp)	P	Het		Muscular dystrophy-dystroglycanopathy (limb-girdle), type C5	Described	Described
			c.1012G > T (p.Val338Leu)	VUS	Het		Muscular dystrophy-dystroglycanopathy (limb-girdle), type C5	Described	Described
143	KCNT1	NM_020822.3	c.1136G > A (p.Ser379Asn)	VUS	Het		Developmental and epileptic encephalopathy, 14	Not Described	Described
144	MCCC2	NM_022132.5	c.1322T > C (p.Ile441Thr)	VUS	Hom		Methylcrotonyl-CoA carboxylase deficiency	Described	Described
	ERCC8	NM_000082.3	c.763A > G (p.Ser255Gly)	VUS	Hom		Cockayne syndrome type A	Not Described	Not Described
145	MMACHC	NM_000082.3	c.8C > G (p.Pro3Arg)	VUS	Hom		Cobalamin C disease	Not Described	Described
148	DNM1	NM_001288739.1	c.2468C > T (p.Pro823Leu)	VUS	Het		Early infantile epileptic encephalopathy type 31	Not Described	Not Described
149	MECP2	NM_001110792.2	c.62 + 1G > A	LP	Het		Rett syndrome	Described	Described
150	CDON	NM_001243597.1	c.1313_1316dup (p.Tyr439*)	LP	Het		Holoprosencéphalie type 11	Not Described	Not Described
151	GRIN2D	NM_000836.2	c.220_221delinsAG (p.Ala74Arg)	VUS	Het		Childhood epileptic encephalopathy type 46	Not Described	Not Described
152	OCRL	NM_001318784.2	c.2086C > T (p.Arg696*)	P	Hem		Lowe syndrome	Not Described	Described
153	COL6A2	NM_001849.3	c.2489G > A (p.Arg830Gln)	VUS	Hom		COL6A2-associated myopathy	Described	Described
154	KCNA2	NM_004974.3	c.539_541del (p.Phe180del)	VUS	Het		Early infantile epileptic encephalopathy type 32	Not Described	Not Described
155	PTH	NM_001316352.1	c.110A > C (p.Lys37Thr)	VUS	Het		Hypoparathyroïdie	Not Described	Not Described
156	COQ8A	NM_020247.5	c.1506 + 1G > A	LP	Hom		Coenzyme Q10 deficiency type 4	Described	Described
157	JMJD1C	NM_032776.2	c.6251C > T (p.Ala2084Val)	VUS	Het	DE NOVO	Central Nervous System Germinoma	Not Described	Not Described
158	MTOR	NM_004958.3	c.5365-5A > G	VUS	Het		Smith-Kingsmore syndrome	Not Described	Described
159	CPA6	NM_020361.4	c.268G > T (p.Gly90Cys)	VUS	Het		Epilepsy temporal familial type 5	Not Described	Not Described
160	HPRT1	NM_000194.3	c.384 + 1G > A	P	Hem		Lesch-Nyhan syndrome	Not Described	Described
161	ADD3	NM_001320591.1	c.1100G > A (p.Gly367Asp)	P	Hom		Spastic Tetraplegia Cerebral Palsy Type 3	Described	Described
163	LAMA2	NM_000426.4	c.8244 + 1G > A	P	Hom		LAMA2-related muscular dystrophy	Described	Described
165	DMD	NM_004006.3	c.10171C > T (p.Arg3391*)	P	Hem	DE NOVO	Duchenne or Becker muscular dystrophy	Described	Described
	NLGN4X	NM_001282145.1	c.2330A > G (p.Asn777Ser)	VUS	Hem	AR	NLGN4X-related X-linked Mental retardation	Not Described	Described
166	CACNA1S	NM_000069.3	c.1583G > A (p.Arg528His)	P	Het		Hypokalemic periodic paralysis type 1	Described	Described
170	HARS	NM_002109.5	c.727A > G (p.Lys243Glu)	VUS	Het		Charcot-Marie-Tooth disease	Not Described	Not Described
171	TPP1	NM_000391.4	c.1094G > A (p.Cys365Tyr)	P	Hem		Neuronal ceroid lipofuscinosis 2	Described	Described
173	NLGN3	NM_181303.2	c.2222T > C (p.Leu741Pro)	VUS	Hem		Autism Spectrum Disorder	Not Described	Not Described
174	KMT2A	NM_001197104.1	c.2343del (p.Ser781Argfs*12)	LP	Het		Wiedemann-Steiner syndrome	Not Described	Not Described
175	SGCA	NM_000023.3	c.100C > T (p.Arg34Cys)	LP	Hom		Autosomal recessive limb-girdle muscular dystrophy type 2D	Described	Described
176	KCNB1	NM_004975.4	c.629C > T (p.Thr210Met)	LP	Het	DE NOVO	Early infantile epileptic encephalopathy type 26 and Pitt-Hopkins syndrome	Described	Described
177	FOXG1	NM_005249.4	c.1021dup (p.Tyr341Leufs*114)	LP	Het		Rett syndrome	Not Described	Not Described
179	MED13L	NM_015335.4	c.5734T > C (p.Trp1912Arg)	LP	Het	DE NOVO	Mental Retardation and Distinctive Facial Features	Not Described	Not Described
180	GNB1	NM_001282539.1	c.281C > G (p.Pro94Arg)	VUS	Het		Autosomal dominant mental retardation, autosomal dominant 42 disorders	Not Described	Described
	KIF1A	NM_001244008.1	c.2467G > C (p.Glu823Gln)	VUS	Het		Autosomal dominant mental retardation, autosomal dominant 9 disorders	Not Described	Not Described
181	TBR1	NM_006593.4	c.107A > G (p.Asp36Gly)	VUS	Het		Autosomal dominant intellectual developmental disorder with autism and speech delay	Not Described	Not Described
182	GAN	NM_022041.4	c.994G > A (p.Gly332Arg)	VUS	Hom		Autosomal recessive giant axonal neuropathy type 1	Described	Described
186	KCNQ2	NM_172107.2	c.544G > A (p.Val182Met)	VUS	Het		Autosomal dominant KCNQ2-related disorders	Described	Described
	SETBP1	NM_015559.2	c.1790C > T (p.Thr597Ile)	VUS	Het		Autosomal dominant mental retardation type 29	Not Described	Not Described
187	NLGN4X	NM_001282145.1	c.502G > A (p.Val168Ile)	VUS	Hem		X-linked NLGN4X-related susceptibility to autism disorders	Not Described	Not Described

*P: Pathogenic/ LP: Likely pathogenic/ VUS: Variant of uncertain significance/ Hom: Homozygous/ Het: Heterozygous/ AD: Autosomal dominant/ AR: Autosomal recessive.

Among the identified SNVs, 65 had been previously documented in scientific literature, 86 were listed in the ClinVar database, and 63 SNVs had no prior record in either the literature or the ClinVar database. We were also able to identify inherited variants in 39 individuals, including four autosomal dominant (AD) variants, ten de novo variants, and 33 autosomal recessive (AR) variants (Table 3).

A total of 123 genes were implicated in this group of patients, encompassing all variants, including those classified as VUS. These genes were associated with neurological pathologies but involved in different pathways (S2 Table), including metabolic pathways, neurodegenerative pathways, and immune-related Pathways. RNASEH2B and LAMA2 were the most frequently identified genes in this study. RNASEH2B, linked to Aicardi-Goutières syndrome 2, was found in three patients who shared the same mutation and exhibited similar clinical features, including pyramidal syndrome, dystonia, and psychomotor delay. LAMA2, associated with congenital muscular dystrophy (alpha subunit-related) and LAMA2-related muscular dystrophy, was found in three patients. Two of these patients shared the same mutation, while the third carried a different mutation. All three exhibited comparable clinical features, including facial dysmorphia, elevated creatine kinase levels, and generalized hypotonia. Thirteen genes were each implicated in two patients: ADD3, CFTR, DNM1, HEXA, IQSEC2, IRF2BPL, KCNA2, NLGN4X, OCRL, PLA2G6, STXBP1, TPP1, and TUBB4A (S3 Table). Additionally 108 genes were each identified in one patient. Each identified variant was analyzed in correlation with the observed phenotype and the specific mutation found in each gene. This analysis allowed us to link the genetic alterations to suspected pathologies potentially underlying the symptoms observed in the patients. For instance, each implicated gene was associated with a distinct pathology, providing insights into its functional role in disease mechanisms (Table 3).

**Fig 3:**
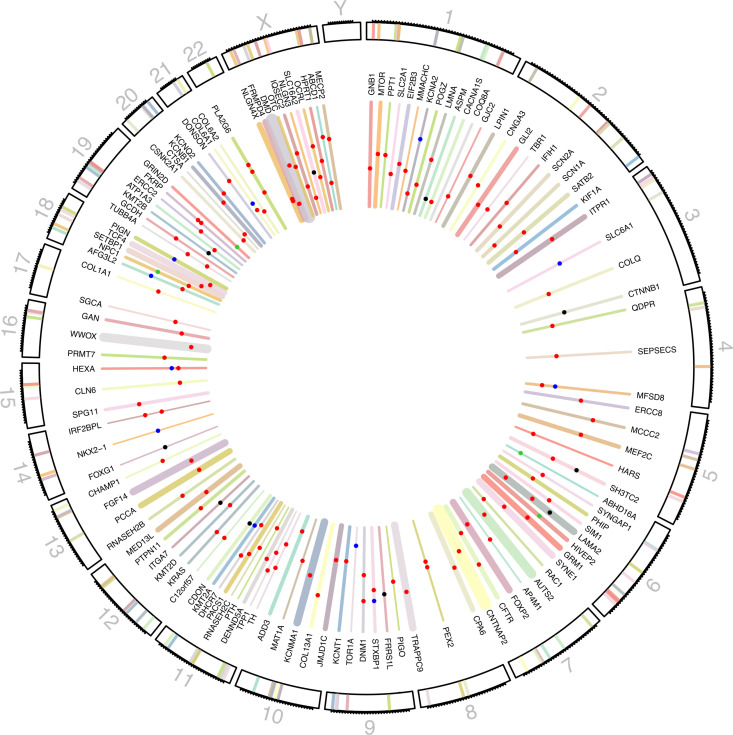
Circular plot representing the spatial distribution of 123 genes and 150 mutations across the genome. Bar thickness represent gene sizes; Mutation types indicated by colors: Red for substitutions, blue for deletions, black for duplications, and green for delins (deletion and insertion). (The scale of chromosome 19 was duplicated for better visibility).

To better visualize the genomic organization and precisely locate variants within specific genes, a circular plot was generated. This visualization illustrates the distribution of the genes and the variants identified in this study. Chromosomes are displayed in the outer ring, while genes are positioned according to the chromosomes they belong to based on their genomic coordinates. Gene size is represented by the thickness of the line in the inner ring. Mutations were integrated into the plot, with distinct colors indicating different mutation types: red for substitution mutations, blue for deletions, black for duplications, and green for delins (deletion and insertion) (Fig 3).

## Discussion

The introduction of WES into medicine has revolutionized how physicians trace the genetic causes of suspected hereditary disorders. WES has recently been adopted in the diagnosis of Mendelian disorders, showing considerable success in identifying rare and genetically diverse conditions [17]. In this study, we present genetic findings from a large group of Moroccan children with undiagnosed or challenging pediatric neurological conditions that remained elusive despite standard testing. Using WES while also detailing the clinical profiles of each patient. Our study achieved a diagnostic yield of 45% (85/188), successfully identifying the genetic causes of various neurological disorders. To our knowledge, no whole exome sequencing studies on pediatric neurological diseases have yet been conducted in North Africa. However, a study from South Africa, which utilized a targeted gene panels rather than whole exome sequencing, reported an overall diagnostic yield of 45% (56/124 patients) [18]. Other studies have reported comparable diagnostic yields for exome sequencing in pediatric neurological disorders. For instance, a study from Saudi Arabia reported a diagnostic yield of 73% (19/26) [19],an Indonesian study documented a 45% yield (9/20) [20], a study from Argentina reported a 40% yield (16/40) [21], and a study from the United States observed a 41% diagnostic yield (32/78) in a heterogeneous pediatric population [22]. By streamlining the diagnostic process, WES reduces the time these patients spend waiting for answers, putting an end to years-long diagnostic journeys, improving their medical management, and enhancing genetic counseling for their families [21].

The relatively high diagnostic yield in our study likely reflects the patient population served by the Rabat Children’s Hospital, a level 3 medical facility that provides highly specialized and advanced care, typically for patients with complex pathologies or requiring advanced interventions. As the top tier in the healthcare hierarchy, following primary care centers (level 1) and secondary hospitals (level 2). It also explains the inter-health heterogeneity observed as WES revealed a wide range of etiologies, including neurodevelopmental, neurodegenerative, epileptic, and neurometabolic disorders among others.

Our study revealed important insights regarding the effectiveness of WES in detecting genetic variants across different clinical phenotypes. Specifically, we found that patients presenting with language disorders had a significantly lower probability of yielding a positive genetic result from WES. This suggests that language disorders alone may not be as strongly linked to the detection of genetic mutations.

In contrast, certain clinical features appeared to have a much stronger association in our study with the identification of diagnostic genetic variants in pediatric patients with neurological diseases, though these associations were not statistically significant. Patients diagnosed with intellectual disability (ID) were among the most likely to have a genetic variant detected, suggesting a strong genetic underpinning for this condition in many cases. Similarly, facial dysmorphia, a characteristic often linked to underlying genetic syndromes, also showed a correlation with positive findings from WES. Furthermore, patients who had a family history of similar clinical presentations were found to be more likely to receive a diagnostic result through genetic testing. These findings underscore the importance of considering a broader clinical and familial context when using WES to identify genetic causes of disease. When compared to previous epilepsy-specific studies [23], which reported statistically significant associations between genetic findings and clinical features like intellectual disability and motor impairments, our study presents a broader perspective. Unlike these study, our findings did not achieve statistical significance. This discrepancy may be explained by the narrower scope of epilepsy-focused research, which concentrates on a single etiology and thus benefits from enhanced statistical power.

The analysis of the 123 genes identified in our study revealed a diverse range of pathways, emphasizing the complex interplay between neurological and non-neurological systems. Some pathways, such as those involved in spinocerebellar ataxia and cholinergic synapse signaling, are directly implicated in neurodegenerative conditions [24]. Others, however, are linked to broader biological processes, such as lysosomal function and metabolic regulation, which are associated with both neurodevelopmental and neurodegenerative disorders [25]. For example, lysosomal storage diseases often result in pediatric neurological impairments like ataxia or cognitive deficits due to disrupted cellular metabolism [26]. Additionally, cardiac-related pathways, including cardiomyopathy and hypertrophic cardiomyopathy, may indirectly affect brain function by impairing circulatory efficiency [27]. These findings highlight the significant overlap between metabolic, immune, and neuronal dysfunctions in pediatric neurological diseases, illustrating the multifactorial nature of these conditions and the interconnectedness of systemic and neurological health. In this study, we also identified a high number of VUS, accounting for 38,5% of the identified variants, with 43 patients presenting exclusively with VUS. Among these patients, 30 exhibited a phenotype-genotype correlation, if these variants are later confirmed as pathogenic, they could significantly increase the diagnostic yield. This observation emphasizes the need for continued efforts to evaluate and reclassify VUS in order to enhance diagnostic accuracy [28]. These findings emphasize the challenges of interpreting such variants, particularly in underrepresented populations such as North Africans and the Middle East, where limited genomic data are published [29,30]. Efforts to compare our findings with other regional data, such as those from Egypt or neighboring countries, will be crucial for improving variant classification and improving diagnostic precision in these populations. In this study, parental testing was not conducted in all cases, which could have provided valuable insight into the inheritance patterns of the identified mutations. And a larger sample size would have been beneficial for better assessment of diagnostic yield and genetic mutation spectrum in pediatrics neurological disorders.

Despite this, we also observed a significant number of pathogenic and likely pathogenic variants, which are highly valuable for clinical diagnostics. These variants were instrumental not only in confirming clinical hypotheses but also in refining and, in some cases, redirecting diagnostic conclusions. In this study, we observed that in some cases, clinical suspicions were concordant with the genetic findings. However, genetic testing not only confirmed the diagnosis but also provided greater precision regarding the underlying cause. For example, in one patient initially suspected of having congenital muscular dystrophy, genetic analysis confirmed a LAMA2-related muscular dystrophy, offering a more specific etiological diagnosis.

In some other cases, the clinical suspicion pointed towards one condition, but genetic results revealed a different diagnosis that still explained the patient’s phenotype. One such case involved a suspected myopathy, but genetic testing identified Paroxysmal dystonic choreoathetosis with epileptic ataxia, an autosomal dominant condition, which better accounted for the patient’s clinical presentation.

We also encountered situations where the clinical hypothesis was not entirely inaccurate, but the genetic diagnosis offered a more refined classification. For instance, in a case initially suspected to be a mitochondrial disorder, genetic analysis revealed a glutaryl-CoA dehydrogenase deficiency. Although this condition is not a mitochondrial disorder in the strict sense, as seen with classical respiratory chain deficiencies, it is an organic aciduria with mitochondrial involvement. The deficient enzyme is located in the mitochondria, and thus, the condition is sometimes included in broader classifications of disorders with secondary mitochondrial implications.

These findings not only provide critical insights into the genetic underpinnings of the conditions studied, but also have a profound psychological impact. Parents of affected children often express relief and reassurance upon receiving a definitive diagnosis, as it provides clarity regarding the cause of their child’s symptoms and facilitates informed decision-making for future care and management [23]. Moreover, WES contributes to reducing unnecessary healthcare costs by enabling faster and more accurate diagnoses, thereby avoiding multiple unrelated and costly diagnostic procedures. This targeted approach enhances overall cost-effectiveness and improves the healthcare journey for families [31]. Looking ahead, future efforts should focus on exploring how to adapt therapeutic approaches now that we have a clearer understanding of the genetic diagnoses in these patients.

## Conclusion

In this study, we investigated the genetic basis of pediatric neurological disorders within a Moroccan hospital-based group of patients, emphasizing the seamless integration of clinical care and research. Our methodology combined detailed phenotyping, supported by up-to-date clinical insights, with the application of current genomic analysis standards and expertise in interpreting genetic variants. This comprehensive approach enhanced our ability to identify genetic contributions to these conditions, improving diagnostic outcomes. Expanding the implementation of these genetic approaches in routine diagnostic workflows could further refine precision and patient management for individuals with neurological disorders, offering a transformative potential for clinical practice.

## Supporting information

S1 Table The patient’s epidemiologic, clinical, and paraclinical data. (XLSX)

S2 Table Kegg pathways of the 123 genes identified in the study. (DOCX)

S3 Table Recurrent genetic findings among cases. (DOCX)

## References

[1] Ogundele MO, Morton M. Classification, prevalence and integrated care for neurodevelopmental and child mental health disorders: a brief overview for paediatricians. World J Clin Pediatr. 2022;11(2):120–35. 35433298 10.5409/wjcp.v11.i2.120 PMC8985496

[2] Savatt JM, Myers SM. Genetic Testing in Neurodevelopmental Disorders. Front Pediatr. 2021;9:526779. doi: 10.3389/fped.2021.526779 33681094 PMC7933797

[3] van Nimwegen KJM, Schieving JH, Willemsen MAAP, Veltman JA, van der Burg S, van der Wilt GJ, et al. The diagnostic pathway in complex paediatric neurology: a cost analysis. Eur J Paediatr Neurol. 2015;19(2):233–9. doi: 10.1016/j.ejpn.2014.12.014 25604808

[4] Fogel BL, Satya-Murti S, Cohen BH. Clinical exome sequencing in neurologic disease. Neurol Clin Pract. 2016;6(2):164–76. doi: 10.1212/CPJ.0000000000000239 27104068 PMC4828678

[5] Sun H, Shen X-R, Fang Z-B, Jiang Z-Z, Wei X-J, Wang Z-Y, et al. Next-Generation Sequencing Technologies and Neurogenetic Diseases. Life (Basel). 2021;11(4):361. doi: 10.3390/life11040361 33921670 PMC8072598

[6] Fernandez-Marmiesse A, Gouveia S, Couce ML. NGS Technologies as a Turning Point in Rare Disease Research , Diagnosis and Treatment. Curr Med Chem. 2018;25(3):404–32. doi: 10.2174/0929867324666170718101946 28721829 PMC5815091

[7] Li H. Aligning sequence reads, clone sequences and assembly contigs with BWA-MEM. arXiv preprint arXiv:13033997. 2013.

[8] Chen X, Schulz-Trieglaff O, Shaw R, Barnes B, Schlesinger F, Källberg M. Manta: rapid detection of structural variants and indels for germline and cancer sequencing applications. Bioinformatics. 2016;32(8):1220–2. 26647377 10.1093/bioinformatics/btv710

[9] ANNOVAR: functional annotation of genetic variants from high-throughput sequencing data | Nucleic Acids Research | Oxford Academic [Internet]. [cited 2024 Oct 20]. Available from: https://academic.oup.com/nar/article/38/16/e164/1749458 10.1093/nar/gkq603 PMC2938201 20601685

[10] Sherry ST, Ward MH, Kholodov M, Baker J, Phan L, Smigielski EM, et al. dbSNP: the NCBI database of genetic variation. Nucleic Acids Res. 2001;29(1):308–11. doi: 10.1093/nar/29.1.308 11125122 PMC29783

[11] ClinVar: public archive of relationships among sequence variation and human phenotype | Nucleic Acids Research | Oxford Academic [Internet]. [cited 2024 Oct 20]. Available from: https://academic.oup.com/nar/article/42/D1/D980/1051029 10.1093/nar/gkt1113 PMC3965032 24234437

[12] Richards S, Aziz N, Bale S, Bick D, Das S, Gastier-Foster J, et al. Standards and guidelines for the interpretation of sequence variants: a joint consensus recommendation of the American College of Medical Genetics and Genomics and the Association for Molecular Pathology. Genet Med. 2015;17(5):405–24. doi: 10.1038/gim.2015.30 25741868 PMC4544753

[13] Szklarczyk D, Gable A, Lyon D, Junge A, Wyder S, Huerta-Cepas J. String v11: protein-protein association networks with increased coverage, supporting functional discovery in genome-wide experimental datasets. Nucleic Acids Res. 2019;47(D1):D607–13. 10.1093/nar/gky1131 PMC6323986 30476243

[14] Kanehisa M. The KEGG Database. In: ‘In Silico’ Simulation of Biological Processes [Internet]. John Wiley & Sons, Ltd; 2002 [cited 2024 Nov 25]. p. 91–103. Available from: https://onlinelibrary.wiley.com/doi/abs/10.1002/0470857897.ch8

[15] Croft D, O’Kelly G, Wu G, Haw R, Gillespie M, Matthews L, et al. Reactome: a database of reactions, pathways and biological processes. Nucleic Acids Res. 2011;39:D691–7. doi: 10.1093/nar/gkq1018 21067998 PMC3013646

[16] Gene Ontology Consortium. The gene ontology (GO) database and informatics resource. Nucleic Acids Res. 2004;32(suppl_1):D258–61. 10.1093/nar/gkh036 PMC308770 14681407

[17] Vinkšel M, Writzl K, Maver A, Peterlin B. Improving diagnostics of rare genetic diseases with NGS approaches. J Community Genet. 2021;12(2):247–56. doi: 10.1007/s12687-020-00500-5 33452619 PMC8141085

[18] Utility of next generation sequencing in paediatric neurological disorders: experience from South Africa | European Journal of Human Genetics [Internet]. [cited 2025 Jan 26]. Available from: https://www.nature.com/articles/s41431-024-01582-2 10.1038/s41431-024-01582-2 PMC11499987 38702429

[19] Muthaffar OY. The Utility of Whole Exome Sequencing in Diagnosing Pediatric Neurological Disorders. Balkan J Med Genet. 2021;23(2):17–24. doi: 10.2478/bjmg-2020-0028 33816068 PMC8009565

[20] Triono A, Iskandar K, Hadiyanto ML, Nugrahanto AP, Diantika K, Wijayanti VW, et al. Identification of the genetic basis of pediatric neurogenetic disorders at a tertiary referral hospital in Indonesia: Contribution of whole exome sequencing. PLoS One. 2023;18(10):e0293113. doi: 10.1371/journal.pone.0293113 37878632 PMC10599538

[21] Córdoba M, Rodriguez-Quiroga SA, Vega PA, Salinas V, Perez-Maturo J, Amartino H, et al. Whole exome sequencing in neurogenetic odysseys: An effective, cost- and time-saving diagnostic approach. PLoS One. 2018;13(2):e0191228. doi: 10.1371/journal.pone.0191228 29389947 PMC5794057

[22] Srivastava S, Cohen JS, Vernon H, Barañano K, McClellan R, Jamal L, et al. Clinical whole exome sequencing in child neurology practice. Ann Neurol. 2014;76(4):473–83. doi: 10.1002/ana.24251 25131622

[23] Koh HY, Smith L, Wiltrout KN, Podury A, Chourasia N, D’Gama AM, et al. Utility of Exome Sequencing for Diagnosis in Unexplained Pediatric-Onset Epilepsy. JAMA Netw Open. 2023;6(7):e2324380. doi: 10.1001/jamanetworkopen.2023.24380 37471090 PMC10359957

[24] Moro A, Moscovich M, Farah M, Camargo CHF, Teive HAG, Munhoz RP. Nonmotor symptoms in spinocerebellar ataxias (SCAs). Cerebellum Ataxias. 2019;6:12. doi: 10.1186/s40673-019-0106-5 31485334 PMC6712685

[25] Full article: An overview of inborn errors of metabolism affecting the brain: from neurodevelopment to neurodegenerative disorders [Internet]. [cited 2024 Nov 22]. Available from: https://www.tandfonline.com/doi/full/10.31887/DCNS.2018.20.4/jmsaudubray 10.31887/DCNS.2018.20.4/jmsaudubray PMC6436954 30936770

[26] Platt FM, d’Azzo A, Davidson BL, Neufeld EF, Tifft CJ. Lysosomal storage diseases. Nat Rev Dis Primers. 2018;4(1):1–25. 30275469 10.1038/s41572-018-0025-4

[27] Arrhythmogenic right ventricular cardiomyopathy: evaluation of the current diagnostic criteria and differential diagnosis | European Heart Journal | Oxford Academic [Internet]. [cited 2024 Nov 22]. Available from: https://academic.oup.com/eurheartj/article/41/14/1414/5602183 10.1093/eurheartj/ehz669 PMC7138528 31637441

[28] A multicenter study of clinical impact of variant of uncertain significance reclassification in breast, ovarian and colorectal cancer susceptibility genes - Makhnoon - 2023 - Cancer Medicine - Wiley Online Library [Internet]. [cited 2024 Dec 14]. Available from: https://onlinelibrary.wiley.com/doi/10.1002/cam4.5202 10.1002/cam4.5202 PMC9939195 36426404

[29] Population Genome Programs across the Middle East and North Africa: Successes, Challenges, and Future Directions | Biomedicine Hub | Karger Publishers [Internet]. [cited 2024 Nov 22]. Available from: https://karger.com/bmh/article/8/1/60/854242/Population-Genome-Programs-across-the-Middle-East 10.1159/000530619 PMC10601860 37900972

[30] known unknown: the challenges of genetic variants of uncertain significance in clinical practice | Journal of Law and the Biosciences | Oxford Academic [Internet]. [cited 2024 Nov 22]. Available from: https://academic.oup.com/jlb/article/4/3/648/4820755 10.1093/jlb/lsx038 PMC5965500 29868193

[31] Chung CCY, Leung GKC, Mak CCY, Fung JLF, Lee M, Pei SLC, et al. Rapid whole-exome sequencing facilitates precision medicine in paediatric rare disease patients and reduces healthcare costs. Lancet Reg Health West Pac. 2020;1:100001. doi: 10.1016/j.lanwpc.2020.100001 34327338 PMC8315561

