# Supplementary material for: Unraveling genetic etiologies in complex pediatric neurological diseases: A genetic investigation using whole exome sequencing
Source: PLoS One. 2025 May 19;20(5):e0324177. doi: 10.1371/journal.pone.0324177 (PMC12088513; doi:10.1371/journal.pone.0324177)
Supplement: S2 Table — (DOCX) [file pone.0324177.s002.docx]

**Table S2.** Kegg pathways of the 123 genes identified in the study.

| **#Term ID** | **Term description** | **Observed gene count** | **Background gene count** | **Strength** | **Signal** | **False discovery rate** | **Matching proteins in the network (IDs)** | **Matching proteins in the network (labels)** |
| --- | --- | --- | --- | --- | --- | --- | --- | --- |
| hsa05412 | Arrhythmogenic right ventricular cardiomyopathy | 7 | 77 | 1.16 | 0.89 | 0.00031 | 9606.ENSP00000262018,9606.ENSP00000354923,9606.ENSP00000355192,9606.ENSP00000357283,9606.ENSP00000400365,9606.ENSP00000452120,9606.ENSP00000495360 | SGCA,DMD,CACNA1S,LMNA,LAMA2,ITGA7,CTNNB1 |
| hsa04142 | Lysosome | 7 | 125 | 0.95 | 0.65 | 0.0024 | 9606.ENSP00000269228,9606.ENSP00000299427,9606.ENSP00000361562,9606.ENSP00000403663,9606.ENSP00000455114,9606.ENSP00000493153,9606.ENSP00000493218 | NPC1,TPP1,CTSA,AP4M1,HEXA,PPT1,MFSD8 |
| hsa04919 | Thyroid hormone signaling pathway | 7 | 120 | 0.97 | 0.65 | 0.0024 | 9606.ENSP00000256078,9606.ENSP00000281928,9606.ENSP00000354558,9606.ENSP00000416293,9606.ENSP00000444688,9606.ENSP00000465734,9606.ENSP00000495360 | KRAS,MED13L,MTOR,SLC2A1,ATP1A3,SLC16A2,CTNNB1 |
| hsa05410 | Hypertrophic cardiomyopathy | 6 | 88 | 1.04 | 0.66 | 0.0024 | 9606.ENSP00000262018,9606.ENSP00000354923,9606.ENSP00000355192,9606.ENSP00000357283,9606.ENSP00000400365,9606.ENSP00000452120 | SGCA,DMD,CACNA1S,LMNA,LAMA2,ITGA7 |
| hsa05414 | Dilated cardiomyopathy | 6 | 94 | 1.01 | 0.65 | 0.0025 | 9606.ENSP00000262018,9606.ENSP00000354923,9606.ENSP00000355192,9606.ENSP00000357283,9606.ENSP00000400365,9606.ENSP00000452120 | SGCA,DMD,CACNA1S,LMNA,LAMA2,ITGA7 |
| hsa01100 | Metabolic pathways | 21 | 1435 | 0.37 | 0.37 | 0.0131 | 9606.ENSP00000039007,9606.ENSP00000281243,9606.ENSP00000298556,9606.ENSP00000301067,9606.ENSP00000326570,9606.ENSP00000333142,9606.ENSP00000343657,9606.ENSP00000347717,9606.ENSP00000360154,9606.ENSP00000361287,9606.ENSP00000365462,9606.ENSP00000367880,9606.ENSP00000370571,9606.ENSP00000371535,9606.ENSP00000397908,9606.ENSP00000398837,9606.ENSP00000436786,9606.ENSP00000455114,9606.ENSP00000466845,9606.ENSP00000492233,9606.ENSP00000493153 | OTC,QDPR,HPRT1,KMT2D,FKRP,PLA2G6,MCCC2,DHCR7,OCRL,MAT1A,PCCA,PIGO,TH,SEPSECS,LPIN1,KMT2B,KMT2A,HEXA,GCDH,PIGN,PPT1 |
| hsa04512 | ECM-receptor interaction | 5 | 88 | 0.96 | 0.48 | 0.0131 | 9606.ENSP00000225964,9606.ENSP00000300527,9606.ENSP00000355180,9606.ENSP00000400365,9606.ENSP00000452120 | COL1A1,COL6A2,COL6A1,LAMA2,ITGA7 |
| hsa05017 | Spinocerebellar ataxia | 6 | 135 | 0.85 | 0.47 | 0.0131 | 9606.ENSP00000263269,9606.ENSP00000269143,9606.ENSP00000282753,9606.ENSP00000306253,9606.ENSP00000354558,9606.ENSP00000365301 | GRIN2D,AFG3L2,GRM1,ITPR1,MTOR,FGF14 |
| hsa04974 | Protein digestion and absorption | 5 | 100 | 0.9 | 0.44 | 0.0186 | 9606.ENSP00000225964,9606.ENSP00000300527,9606.ENSP00000355180,9606.ENSP00000381949,9606.ENSP00000444688 | COL1A1,COL6A2,COL6A1,COL13A1,ATP1A3 |
| hsa00310 | Lysine degradation | 4 | 60 | 1.03 | 0.43 | 0.0226 | 9606.ENSP00000301067,9606.ENSP00000398837,9606.ENSP00000436786,9606.ENSP00000466845 | KMT2D,KMT2B,KMT2A,GCDH |
| hsa04720 | Long-term potentiation | 4 | 63 | 1.01 | 0.43 | 0.0226 | 9606.ENSP00000256078,9606.ENSP00000263269,9606.ENSP00000282753,9606.ENSP00000306253 | KRAS,GRIN2D,GRM1,ITPR1 |
| hsa04725 | Cholinergic synapse | 5 | 109 | 0.87 | 0.42 | 0.0226 | 9606.ENSP00000256078,9606.ENSP00000306253,9606.ENSP00000352035,9606.ENSP00000355192,9606.ENSP00000367872 | KRAS,ITPR1,KCNQ2,CACNA1S,GNB1 |
| hsa05165 | Human papillomavirus infection | 8 | 324 | 0.6 | 0.36 | 0.0286 | 9606.ENSP00000225964,9606.ENSP00000256078,9606.ENSP00000300527,9606.ENSP00000354558,9606.ENSP00000355180,9606.ENSP00000400365,9606.ENSP00000452120,9606.ENSP00000495360 | COL1A1,KRAS,COL6A2,MTOR,COL6A1,LAMA2,ITGA7,CTNNB1 |
| hsa04151 | PI3K-Akt signaling pathway | 8 | 349 | 0.56 | 0.34 | 0.0376 | 9606.ENSP00000225964,9606.ENSP00000256078,9606.ENSP00000300527,9606.ENSP00000354558,9606.ENSP00000355180,9606.ENSP00000367872,9606.ENSP00000400365,9606.ENSP00000452120 | COL1A1,KRAS,COL6A2,MTOR,COL6A1,GNB1,LAMA2,ITGA7 |
| hsa04510 | Focal adhesion | 6 | 195 | 0.69 | 0.35 | 0.0376 | 9606.ENSP00000225964,9606.ENSP00000300527,9606.ENSP00000355180,9606.ENSP00000400365,9606.ENSP00000452120,9606.ENSP00000495360 | COL1A1,COL6A2,COL6A1,LAMA2,ITGA7,CTNNB1 |
| hsa05205 | Proteoglycans in cancer | 6 | 194 | 0.69 | 0.35 | 0.0376 | 9606.ENSP00000225964,9606.ENSP00000256078,9606.ENSP00000306253,9606.ENSP00000354558,9606.ENSP00000489597,9606.ENSP00000495360 | COL1A1,KRAS,ITPR1,MTOR,PTPN11,CTNNB1 |
| hsa05010 | Alzheimer disease | 8 | 354 | 0.56 | 0.33 | 0.0377 | 9606.ENSP00000217244,9606.ENSP00000256078,9606.ENSP00000263269,9606.ENSP00000264071,9606.ENSP00000306253,9606.ENSP00000354558,9606.ENSP00000355192,9606.ENSP00000495360 | CSNK2A1,KRAS,GRIN2D,TUBB4A,ITPR1,MTOR,CACNA1S,CTNNB1 |
| hsa04911 | Insulin secretion | 4 | 82 | 0.89 | 0.37 | 0.0378 | 9606.ENSP00000286628,9606.ENSP00000355192,9606.ENSP00000416293,9606.ENSP00000444688 | KCNMA1,CACNA1S,SLC2A1,ATP1A3 |
| hsa04072 | Phospholipase D signaling pathway | 5 | 147 | 0.74 | 0.34 | 0.0440 | 9606.ENSP00000256078,9606.ENSP00000282753,9606.ENSP00000354558,9606.ENSP00000362014,9606.ENSP00000489597 | KRAS,GRM1,MTOR,DNM1,PTPN11 |
| hsa04540 | Gap junction | 4 | 87 | 0.87 | 0.35 | 0.0440 | 9606.ENSP00000256078,9606.ENSP00000264071,9606.ENSP00000282753,9606.ENSP00000306253 | KRAS,TUBB4A,GRM1,ITPR1 |
| hsa05235 | PD-L1 expression and PD-1 checkpoint pathway in cancer | 4 | 87 | 0.87 | 0.35 | 0.0440 | 9606.ENSP00000217244,9606.ENSP00000256078,9606.ENSP00000354558,9606.ENSP00000489597 | CSNK2A1,KRAS,MTOR,PTPN11 |
| hsa04934 | Cushing syndrome | 5 | 153 | 0.72 | 0.33 | 0.0469 | 9606.ENSP00000301067,9606.ENSP00000306253,9606.ENSP00000355192,9606.ENSP00000436786,9606.ENSP00000495360 | KMT2D,ITPR1,CACNA1S,KMT2A,CTNNB1 |
