# Supplementary material for: Unraveling genetic etiologies in complex pediatric neurological diseases: A genetic investigation using whole exome sequencing
Source: PLoS One. 2025 May 19;20(5):e0324177. doi: 10.1371/journal.pone.0324177 (PMC12088513; doi:10.1371/journal.pone.0324177)
Supplement: S3 Table — (DOCX) [file pone.0324177.s003.docx]

**Table S3.** Recurrent genetic findings among cases.

| **Gene** | **Occurrence^a^** | **OMIM description/Pubmed Description** | **OMIM** | **Nb of variants ^b^** | **Pathogenic** | **Likely pathogenic** | **VUS** |
| --- | --- | --- | --- | --- | --- | --- | --- |
| *RNASEH2B* | 3 | Aicardi-Goutieres syndrome 2 | OMIM: 610181 | 1 | 1 | 0 | 0 |
| LAMA2 | 3 | Alpha subunit-related congenital muscular dystrophy/ LAMA2-related muscular dystrophy | OMIM: 156225 | 3 | 2 | 1 | 0 |
| ADD3 | 2 | Spastic Tetraplegia Cerebral Palsy Type 3 | OMIM: 601568 | 1 | 1 | 0 | 0 |
| CFTR | 2 | Cystic Fibrosis /CFTR-related disorder. | OMIM: 602421 | 3 | 3 | 0 | 0 |
| DNM1 | 2 | Developmental and epileptic encephalopathy, 31/ Early infantile epileptic encephalopathy type 31 | OMIM: 602377 | 2 | 0 | 1 | 1 |
| HEXA | 2 | Tay-Sachs disease | OMIM: 606869 | 2 | 1 | 1 | 0 |
| IQSEC2 | 2 | Intellectual disability, X-linked 1 | OMIM: 300522 | 2 | 0 | 1 | 1 |
| IRF2BPL | 2 | Neurodevelopmental disorder with regression, abnormal movements | OMIM: 618088 | 2 | 0 | 1 | 1 |
| KCNA2 | 2 | Early infantile epileptic encephalopathy type 32 | OMIM: 176262 | 2 | 0 | 0 | 2 |
| NLGN4X | 2 | NLGN4X-related X-linked Mental retardation  X-linked NLGN4X-related susceptibility to autism disorders | OMIM: 300427 | 2 | 0 | 0 | 2 |
| OCRL | 2 | Lowe syndrome | OMIM: 300535 | 2 | 2 | 0 | 0 |
| PLA2G6 | 2 | Infantile neuroaxonal dystrophy | OMIM: 603604 | 4 | 3 | 0 | 1 |
| STXBP1 | 2 | Developmental and epileptic encephalopathy, 4  / Early Autosomal Dominant Infantile Epileptic Encephalopathy Type 4 | OMIM: 602926 | 2 | 2 | 0 | 0 |
| TPP1 | 2 | Congenital multiple-hypotonia-epileptic / Neuronal ceroid lipofuscinosis 2 | OMIM: 607998 | 2 | 2 | 0 | 0 |
| TUBB4A | 2 | Leukodystrophy, Hypomyelinating, 6 / Hypomyelination with atrophy of the central gray nuclei | OMIM: 602662 | 2 | 1 | 1 | 0 |
|  |  |  |  |  |  |  |  |

**^a^ gene occurrence in patients
^b^** **Number of variants occurring in the same gene**
